# The diagnostic value of contrast-enhanced transcranial Doppler and contrast-enhanced transthoracic echocardiography for right to left shunt in patent foramen ovale: a systematic review and meta-analysis

**DOI:** 10.3389/fneur.2024.1447964

**Published:** 2024-08-02

**Authors:** Dian Zhang, Li Jiang, Yue-Nan Chen, Mei-Fang Pan

**Affiliations:** Department of Ultrasound, Xiangcheng People's Hospital, Suzhou, China

**Keywords:** contrast transcranial Doppler, contrast transthoracic echocardiography, patent foramen ovale, right to left shunt, meta-analysis

## Abstract

**Purpose:**

To evaluate and compare the diagnostic value of contrast-enhanced transcranial Doppler (c-TCD) and contrast-enhanced transthoracic echocardiography (c-TTE) for right to left shunt (RLS) in patent foramen ovale (PFO) by meta-analysis.

**Methods:**

The literature included in the Cochrane Library, PubMed, and Embase were searched by using “contrast-enhanced transcranial Doppler (c-TCD), contrast-enhanced transthoracic echocardiography (c-TTE), patent foramen ovale (PFO), and right to left shunt (RLS)” as the keywords from inception through April 30, 2024. The diagnostic accuracy research quality assessment tool (QUADAS-2) was used to evaluate the quality of the included literature. The combined sensitivity, specificity, positive likelihood ratio (PLR), negative likelihood ratio (NLR), and Diagnostic odds ratio (DOR) were pooled, and a comprehensive ROC curve analysis was performed. Statistical software StataSE 12.0 and Meta-Disc 1.4 were used for data analysis.

**Results:**

A total of 8,536 articles were retrieved, and 9 articles that met all inclusion criteria were included in this meta-analysis. The meta-analysis results show that the combined sensitivity, specificity, PLR, NLR, DOR, and area under the SROC curve of c-TCD for the diagnose of PFO-RLS were 0.91 (95% CI, 0.88–0.93), 0.87 (95% CI: 0.84–0.91), 6.0 (95% CI, 2.78–12.96), 0.10 (95% CI, 0.06–0.18), 91.61 (95% CI, 26.55–316.10), and 0.9681, respectively; the corresponding values of c-TTE were 0.86 (95% CI, 0.84–0.89), 0.88 (95% CI, 0.84–0.91), 5.21 (95% CI, 2.55–10.63), 0.16 (95% CI, 0.09–0.31), 71.43 (95% CI, 22.85–223.23), and 0.9532. The ROC curve shows that c-TCD has slightly higher diagnostic value for PFO than c-TTE, but there is no significant statistical difference (Z = 0.622, *p* > 0.05). Deek funnel pattern showed no significant publication bias.

**Conclusion:**

Both c-TCD and c-TTE have high diagnostic values for PFO-RLS. However, c-TCD has slightly higher sensitivity and lower specificity in diagnosing PFO-RLS compared to c-TTE.

**Systematic review registration:** identifier [CRD42024544169].

## 1 Introduction

Patent foramen ovale (PFO) is a passage left in the atrial septum of the heart during embryonic development, with a detection rate of approximately 25–30% in adults (1). Especially in young patients with cryptogenic stroke, the incidence rate is higher. Right to left shunt (RLS) refers to the direct blood flow from the venous circulation system to the arterial circulation system through abnormal channels, without filtration through the lungs (2). PFO is currently the most common cause among RLS types, reaching 95% (3). Multiple diseases such as occult stroke, transient ischemic attack, unexplained syncope, and migraine have been confirmed to be associated with PFO-RLS (3–6). At present, the main diagnostic methods for PFO-RLS ultrasound examination were transesophageal echocardiography (TEE), contrast-enhanced transthoracic echocardiography (c-TTE), and contrast-enhanced transcranial Doppler ultrasound (c-TCD) (7). There are differences in the detection of PFO-RLS among these three examination methods. TEE with right heart contrast echocardiography can display the characteristics of the atrial septal structure, size, and shape of the foramen ovale, and is currently the gold standard for diagnosing patent foramen ovale (8). However, due to its time-consuming and invasive nature, this test is unsuitable for screening PFO. The advantages of non-invasive, easy-to-operate, and high reproducibility of c-TCD and c-TTE during use have gradually made them important methods for screening PFO-RLS (9, 10). However, there are significant differences in opinions on which of the two detection methods had a greater advantage in detecting PFO-RLS (11, 12). Therefore, this study conducted a comprehensive systematic review and meta-analysis to compare the diagnostic value of c-TCD and c-TTE for PFO-RLS, to improve the understanding of the clinical application of the two methods and provide a decision-making basis for clinical doctors.

## 2 Materials and methods

### 2.1 Search strategy

We searched PubMed, Embase, and Cochrane Library from inception through April 30, 2024. The following keywords and MeSH terms were used: [“contrast transcranial Doppler” or “c-TCD”] and [“contrast transthoracic echocardiography” or “c-TTE”] and [“patent foramen ovale” or “PFO”] and [“right to left shunt” or “RLS”]. We also performed a manual search to find other potential articles. Two investigators (DZ and LJ) searched online to obtain the original data, and the reference lists of all relevant articles were also scanned. All retrieved citations were exported to Zotero and checked for duplicates.

### 2.2 Inclusion and exclusion criteria

The inclusion criteria of this study were as follows:(1) Clinical cohort study or diagnostic test; (2) English research; (3) All patients were examined by c-TCD and c-TTE; (4) TEE was used as reference standards. If TEE was not the gold standard, then use TEE as a reference to calculate the appropriate parameters (5) All studies can directly or indirectly obtain original data such as true positive (TP), false positive (FP), false negative (FN), true negative values (TN). Exclusion criteria: (1) Abstracts, reviews, or case reports; (2) Repeated publication of data; (3) Incomplete original data.

### 2.3 Data extraction

Two evaluators (LJ and DZ) independently screened the literature according to the inclusion and exclusion criteria of the literature, and finally obtained two copies of data, and then cross-checked the data. If there were different opinions, discuss and negotiate together or ask a third party to help decide. Extract literature data from the data, including the first author, publication year, country, research type, number of cases, average age of patients, ultrasound system parameters, and echo-contrast medium. This study was conducted by the Preferred Reporting Items for Systematic Reviews and Meta-Analyses (PRISMA) guidelines (13), the protocol was registered in the PROSPERO:(CRD42024544169).

### 2.4 Evaluation of research quality

Use the QUADAS-2 scale in Review Manager software (RevMan, version 5.4, Cochrane IMS) to evaluate bias risk and applicability and create a bias risk and applicability assessment diagram. Risk assessment of bias comprises four areas: patient selection, index test, reference standards, and flow and timing; Applicability assessment covers three areas: patient selection, index test, and reference standards. The evaluation results for each field are evenly divided into high-risk, low-risk, and unclear.

### 2.5 Statistical analysis

Meta-DiSc version 1.4 (Universidad Complutense, Madrid, Spain) software was used for meta-analysis. The sensitivity (Sen), specificity (Spe), positive likelihood ratio (LR+), and negative likelihood ratio (LR-) are calculated, and the threshold effect is evaluated using the 95% confidence interval (CI). The summary receiver operating characteristic (SROC) curve and the corresponding area under the curve were determined. Quality evaluation chart and ROC curve were created using Review Manager (RevMan, version 5.4, Cochrane IMS). The heterogeneity of the diagnostic odds ratio (DOR) of each study was analyzed by Meta-Disc 1.4 software. If I2 > 50% or *p* < 0.05 among the included literature, there is a high heterogeneity between the results; I2 < 25% indicates that the heterogeneity between results is small, and 25% ≤ I2 ≤ 50% indicates that the heterogeneity of results is medium; *p* ≥ 0.05 indicates that there is no heterogeneity in the results. If there is heterogeneity, try to explore the source of heterogeneity by using Meta-regression. The sensitivity analysis was carried out by StataSE 12 (Stata Corporation, College Station, TX). Z-test was used to compare the diagnostic value of c-TCD and c-TTE. *p* < 0.05 indicated a statistically significant difference.

## 3 Results

### 3.1 Literature research and screening results

**Figure 1:**
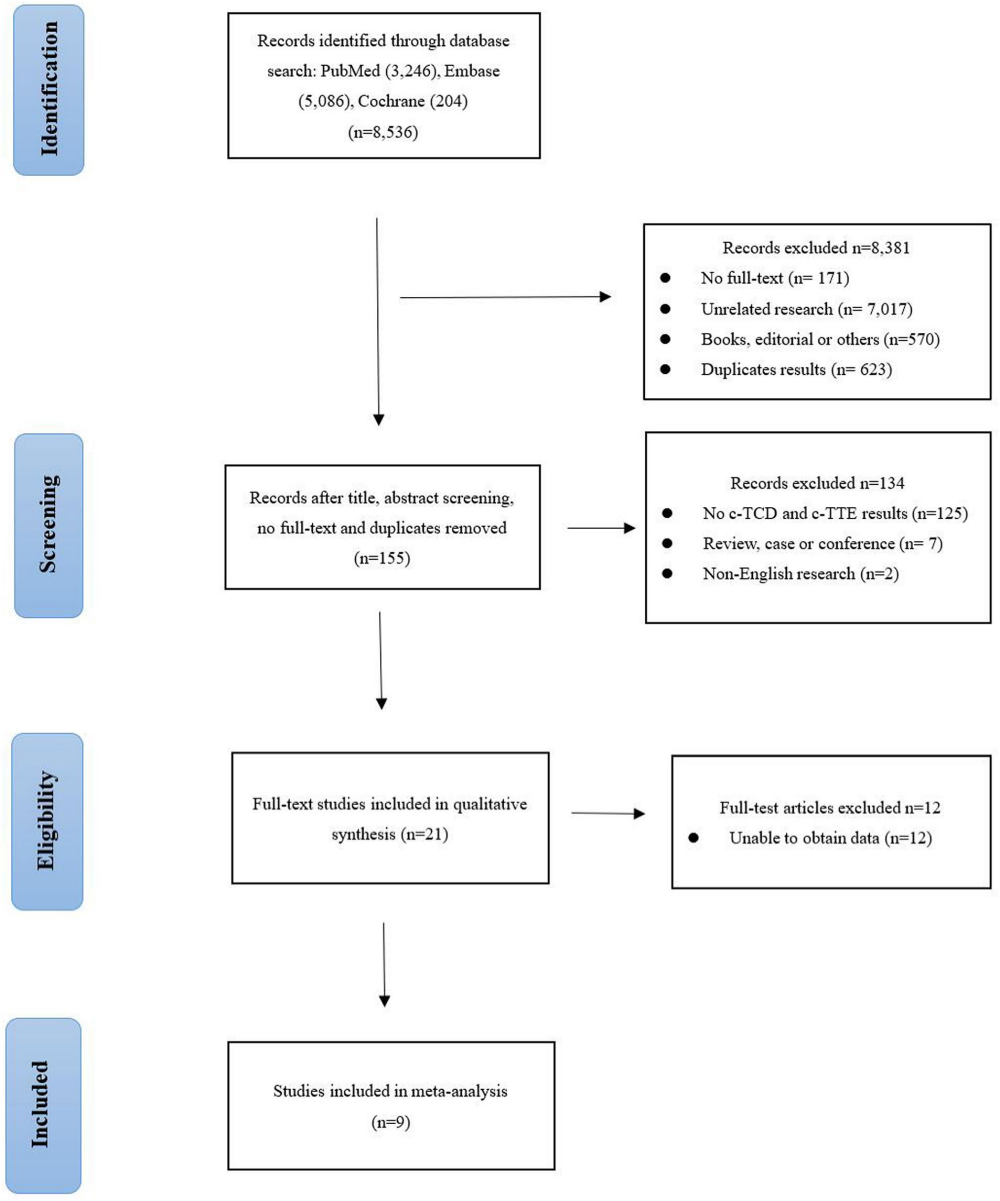
Flowchart of the study selection process.

Figure 1 shows the PRISMA flowchart we studied. After reading the title, the abstract, the full text, and the duplicate articles, 155 articles were selected. After eliminating duplicate results and abstract screening, the number of complete publications that may meet the criteria was reviewed, and finally, 9 articles were included (14–22). Other 146 articles were excluded because of non-c-TCD and c-TTE results, duplication, unrelated research, inappropriate data, review, case, conference, and non-English research.

### 3.2 Basic characteristics and quality assessment of the included literature

The detailed characteristics of the included study are shown in Table 1, including the name of the first author, year of publication, the age range of patients, gender distribution (male/female), design, sample size, echo-contrast medium, and equipment. All these articles were published from 2000 to 2022. The study was conducted in European countries (*n* = 4), China (*n* = 4), and United States (*n* = 1). The sample size is between 72 and 213. A total of 1,086 cases were included in these studies. After excluding invalid nodules, there were 355 non-PFO and 731 PFO cases, respectively. The methodological quality of the included studies based on QUADAS-2. The risk of bias and adherence of individual studies to these items. All included studies had a low risk of bias and were of high quality (Supplementary Figures S1, S2).

**Table 1:** Included studies and the basic characteristics.

Author (Year)	Country	Design	Sex (M/F)	Age (mean ± SD)	PFO/total cases (*n*)	VM position	TCD system	TTE system	Echo-contrast medium
González-Alujas T (2011) (14)	Spain	Prospective	75/59	46.4 ± 14.2	93/134	Left lateral	100 ML system and MultiDop X4	Vivid 7	saline solution and air
Maffè S (2010) (15)	United States	NA	28/47	49 ± 13	62/75	Left lateral	Philips iE33 platform	Philips iE33	saline solution and air
Souteyrand G (2005) (16)	France	NA	67/40	56	42/107	supine	SONOS 5500	SONOS 5500	saline solution and air
Zito C (2009) (17)	Italy	Prospective	33/39	49 ± 13	46/72	Supine	Prosound α-10, ALOKA echo-machine	ALOKA echo-machine	agitated saline solution mixed with urea-linked gelatine
Stendel R (2000) (18)	Germany	Prospective	47/45	51	24/92	Supine	Medasonics CDS	Ultramark 9	D-galactose microspheres and generates air-filled microbubbles
Liu *F* (2020) (19)	China	NA	86/75	42.0 ± 15.6	141/161	Left lateral	Multi-DopX4 Transcranial Doppler	GE Vivid E9 or E95 Philips EPIQ7	saline solution and air
Yang J (2020) (20)	China	NA	68/145	41 ± 12	161/213	–	–	GE or Siemens	saline solution and air
Yang X (2020) (21)	China	Prospective	40/62	41.9 ± 13	98/102	Supine	Vivid 7	Philips Epiq7c	saline, patient’s blood, and room air
Lu J (2022) (22)	China	Retrospective	51/79	c-TCD group:66.32 ± 15.34 c-TTE group:68.80 ± 16.10	64/130	Supine	–	Philips iE	saline, patient’s blood, and room air

VM, valsalva maneuver; PFO, patent foramen ovale; c-TCD, contrast-enhanced transcranial Doppler; c-TTE, contrast-enhanced transthoracic echocardiography; NA, Not Applicable.

### 3.3 Threshold effects and heterogeneity

The Spearman correlation coefficients were 0.433 and 0.733 by heterogeneity analysis (*p* > 0.05), indicating that there was no threshold effect. At the same time, the results showed that heterogeneity for sensitivity (I2 = 78.6%), specificity (I2 = 90%) in the c-TCD group, and sensitivity (I2 = 93.8%), specificity (I2 = 89.1%) in the c-TTE group. The included literature has high heterogeneity, so it is necessary to use a random effect model to summarize and evaluate, and draw SROC curve.

### 3.4 Sensitivity analysis

To observe the stability of the synthetic results, the data included in the literature were excluded one by one and the sensitivity and specificity were summarized again. It showed that the combined effect of various indicators changed little, indicating that the stability of the included literature was good and the reliability of the results was high (Supplementary Figure S3).

### 3.5 Meta-regression analysis

As a result of the significant heterogeneity, meta-regression analysis was used to explore the source of heterogeneity. The covariates of the regression model are set as follows: (1) The sample size ≥100 is set as 1, and the sample size<100 is set as 0; (2) The age ≥ 50 years old is set as 0, and the age < 50 years old is set as 1; (3) The prospective study was set as 0, others study was set at 1; (4) The research object from China is set as 0, and that from other countries is set as 1. Meta-regression analysis of the c-TCD group showed that there was a significant difference between the sources of heterogeneity and age (*p* = 0.03). There was no significant difference between the sources of heterogeneity and the covariates in the c-TTE group (*p* > 0.05).

### 3.6 Diagnostic accuracy

**Figure 2:**
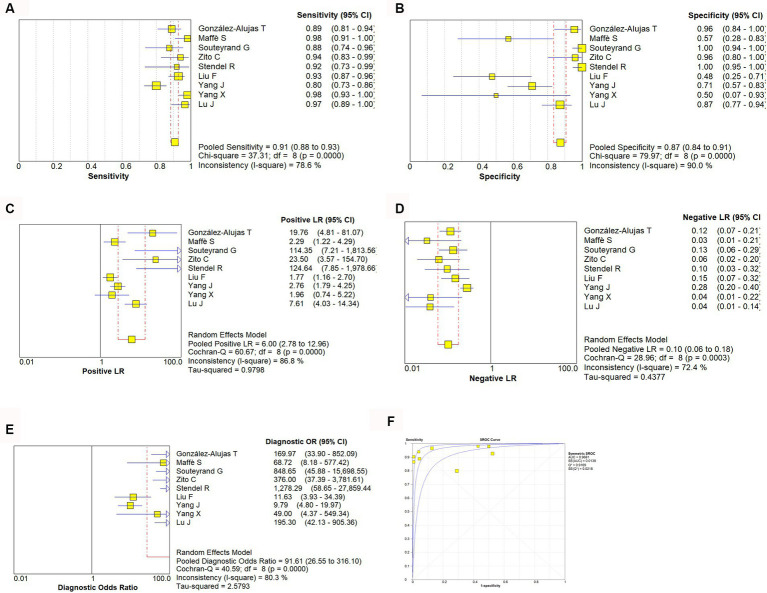
Estimates of c-TCD assessment for the diagnosis of PFO-RLS. **(A–E)** Forest plots illustrate pooled estimates (diamonds) for sensitivity **(A)**, specificity **(B)**, positive likelihood ratio (LR) **(C)**, negative LR **(D)**, and diagnostic odds ratio **(E)** and corresponding 95% CIs for pooled estimates. **(F)** Summary receiver operating characteristic (SROC) plot for assessing accuracy with corresponding curves indicative of upper and lower bounds of 95% CI. AUC, area under curve; SE, standard error; Q*, summary measure of accuracy derived from the SROC curve.

**Figure 3:**
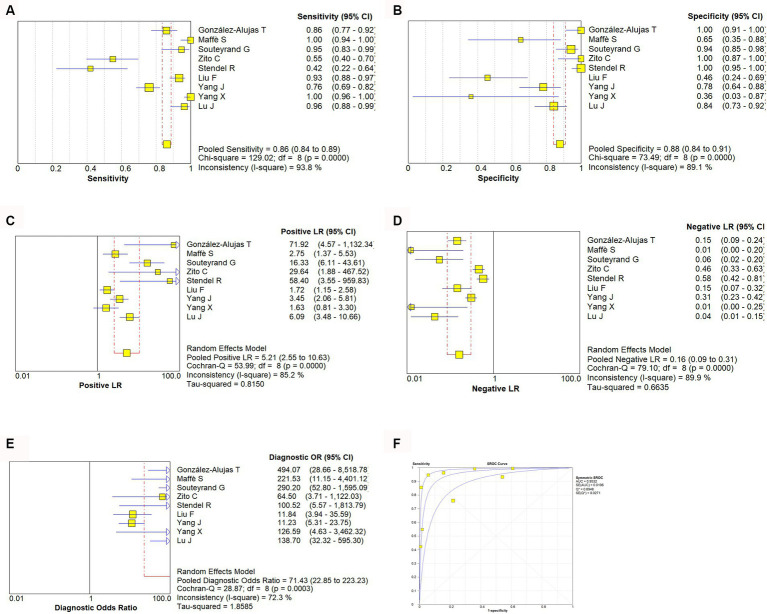
Estimates of c-TTE assessment for the diagnosis of PFO-RLS. **(A–E)** Forest plots illustrate pooled estimates (diamonds) for sensitivity **(A)**, specificity **(B)**, positive likelihood ratio (LR) **(C)**, negative LR **(D)**, and diagnostic odds ratio **(E)** and corresponding 95% CIs for pooled estimates. **(F)** Summary receiver operating characteristic (SROC) plot for assessing accuracy with corresponding curves indicative of upper and lower bounds of 95% CI. AUC, area under curve; SE, standard error; Q*, summary measure of accuracy derived from the SROC curve.

**Figure 4:**
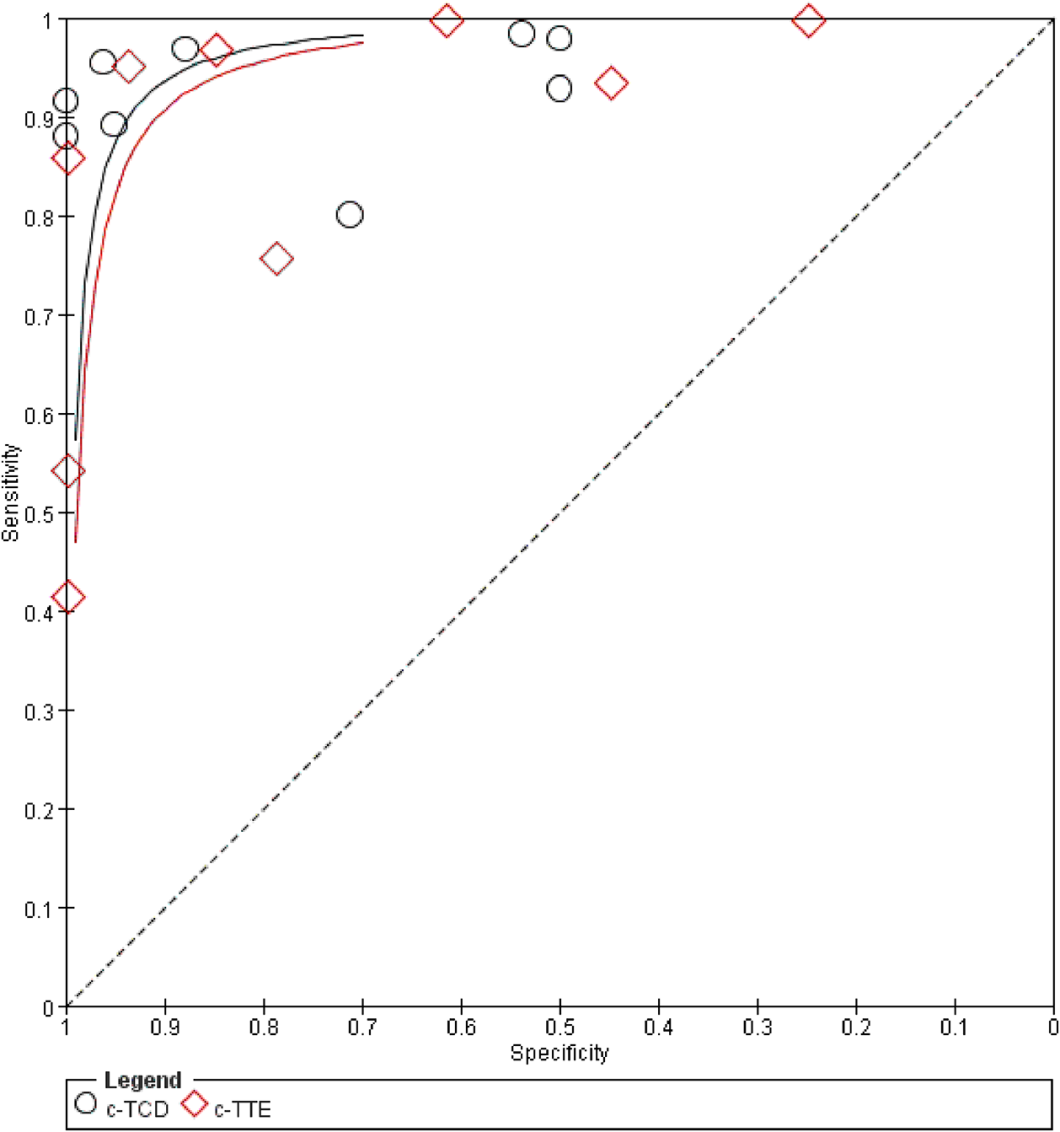
Comparison of ROC curves for the diagnostic value of c-TCD and c-TTE in PFO.

A random effect model was used to analyze the combined effect quantity of the diagnostic four-grid data of c-TCD and c-TTE included in the literature. The combined sensitivity of c-TCD and c-TTE in the diagnosis of PFO-RLS was 0.91 (95% CI: 0.88–0.93) and 0.86 (95% CI: 0.84–0.89), respectively; The combined specificity was 0.87 (95% CI: 0.84–0.91) and 0.88 (95% CI: 0.84–0.91) respectively; The positive likelihood ratios were 6.0 (95% CI: 2.78 ~ 12.96) and 5.21 (95% CI: 2.55 ~ 10.63) respectively; The negative likelihood ratios were 0.10 (95% CI, 0.06 ~ 0.18) and 0.16 (95% CI, 0.09 ~ 0.31) respectively; The DOR were 91.61 (95% CI, 26.55 ~ 316.10) and 71.43 (95% CI, 22.85 ~ 223.23) respectively. The ROC curve shows that c-TCD has slightly higher diagnostic value for PFO than c-TTE. The area under the SROC curve is 0.9681 and 0.9532, respectively. However, there is no significant statistical difference (Z = 0.622, *p* > 0.05) (Figures 2–4).

### 3.7 Evaluation of publication bias and clinical applicability

The meta-analysis of the value of c-TCD and c-TTE showed no significant asymmetry (p > 0.05), that is, there was no significant publication bias (Supplementary Figure S4). At the same time, it can be seen from the Fagan diagram that the post-test probability of c-TCD and c-TTE is 91% respectively, which is 50% higher than the pre-test probability. The combined negative likelihood ratio of c-TCD and c-TTE in the diagnosis of PFO-RLS is more than 0.1, and the combined positive likelihood ratio is less than 10, indicating that both methods are effective in the diagnosis of PFO-RLS (Supplementary Figure S5).

## 4 Discussion

The foramen ovale is an important physiological channel in the embryonic atrial septum. PFO is a dynamic and open channel structure, the pressure in the right atrium is lower than that in the left atrium, and the foramen ovale valve is well-fitted to prevent shunting (23). When the pressure in the left and right atria changes, the foramen ovale valve cannot tightly fit, causing blood to flow between the atria. The direction of shunting depends on the pressure difference between the left and right atria. When the left atrial pressure is lower than the right atrial pressure, the PFO channel opens, and the emboli in the right heart and venous system enter the left heart and arterial system in the opposite direction (23). Research has shown that PFO is closely related to the occurrence of most cerebrovascular diseases (24–26). The reason may be that during deep breathing, severe coughing, or performing the Valsalva maneuver, the right atrial end-diastolic pressure temporarily increases, causing blood clots, air, fat, and vasoactive substances to enter the arterial circulation through the PFO-RLS from the venous circulation. According to statistics, the incidence of PFO in patients with unexplained stroke is much higher than that in the normal healthy population (27). Palazzo found that the prevalence of PFO in patients with cryptogenic stroke ranged from 44 to 66%, while the prevalence of PFO in stroke patients with common causes was only 10 to 27% (28). The detection of PFO has increasingly become a hot topic in clinical research. c-TEE, as the gold standard for diagnosing PFO and RLS, can provide anatomical support for transcatheter PFO occlusion (29, 30). However, during the examination process, patients generally feel uncomfortable and may even cause serious complications such as esophageal perforation and vocal paralysis. In addition, these discomforts can lead to poor coordination of Valsalva movements, resulting in false negatives (8). The principle of c-TCD exploration of PFO-RLS is mainly to inject a microbubble contrast agent into the elbow vein mass and detect at least one side of the middle cerebral artery through TCD to determine whether there is a microembolic signal entering the middle cerebral artery. The principle of c-TTE is to first perform transthoracic echocardiography detection, select the four-chamber view below the xiphoid process, inject microbubble contrast agent through the elbow vein mass, and observe whether microbubbles enter the left atrium. In addition, c-TTE combined with RoPE score can effectively identify high-risk PFO and the probability of related stroke (31). Due to the advantages of simplicity, non-invasive, and low cost, c-TTE and c-TCD are currently routinely performed in most hospitals, but the reliability of these tests is still controversial. Previous research showed that the negative predictive value of c-TCD is greater than that of c-TTE, indicating that c-TCD excludes PFO-RLS better than c-TTE (16). However other research showed that the sensitivity of c-TCD in diagnosing PFO-RLS is lower than that of c-TTE, and the specificity is higher than that of c-TTE (14). Therefore, this study conducted a comprehensive systematic review and meta-analysis to compare the diagnostic value of c-TCD and c-TTE for PFO-RLS, to improve the understanding of the clinical application of the two methods and provide a decision-making basis for clinical doctors.

This study used the diagnostic experimental evaluation tool QUADAS-2 to evaluate the quality of the included literature. The results indicate that the overall quality of the included studies is high and the risk of bias is low. However, the heterogeneity included in the study is significant, but heterogeneity testing indicates the absence of threshold effects, and heterogeneity may be mainly caused by non-threshold effects. This may be due to the characteristics of the patients included in each study, the technical level of the operators, the type of study design, different diagnostic criteria, and different contrast agents. Meta-regression analysis shows that there is a certain relationship between the heterogeneity of c-TCD and age, and the relationship between c-TTE and covariates is not statistically significant. It is speculated that the operator’s dependence on related measurements may also bring some bias. The heterogeneity of this study was not caused by threshold effects; therefore, a random model was used for summary analysis. In addition, this study is a diagnostic meta-analysis, so there is inevitably clinical heterogeneity.

This study found that the combined sensitivity of c-TCD in the diagnosis of PFO-RLS was 91%, specificity was 87%, PLR was 6.0, NLR was 0.10, DOR was 91.61, and the area under the SROC curve (AUC) was 0.9681. The sensitivity and specificity of c-TTE in diagnosing PFO-RLS were 86 and 88%, respectively. The PLR was 5.21, the NLR was 0.16, the DOR was 71.43, and the area under the SROC curve (AUC) was 0.9532. The sensitivity of c-TCD to PFO-RLS detection is higher than that of c-TTE, and the specificity is lower than that of c-TTE. The possible reasons for the analysis may be: The price of c-TCD is cheaper than that of c-TTE, which allows for examination of disabled patients at the bedside, repeated experiments in different positions, and patients are more likely to perform standard Valsalva movements to improve detection sensitivity (32, 33); c-TCD is used to detect both intracardiac and extracardiac RLS, while PFO belong to intracardiac RLS, so specificity is low; The low sensitivity of c-TTE may be due to poor detection image quality, and during Valsalva maneuver, the patient’s chest wall activity is too large, which affects image acquisition and result judgment (34, 35); c-TTE can visually detect the specific conditions of the cardiac structure and surrounding tissues such as the atrial septum, identify the source of RLS, and improve specificity (17). The different techniques used by doctors during operation, the patient’s physical conditions, and the characteristics of testing instruments can all affect the results. For patients with high suspicion of PFO, such as unexplained stroke, migraine, dizziness, and transient ischemic attacks, it is more necessary to consider specificity, while if only clinical screening is used, sensitivity is preferred. In practical clinical work, it is necessary to comprehensively consider the above methods to enable patients to receive more personalized diagnoses and treatment.

The SROC curve is a comprehensive indicator that directly observes the accuracy of diagnostic tests, reflecting the sensitivity and specificity of diagnostic tests and targeted diseases. The AUC value is an important indicator of testing accuracy. The closer to 1, the better the diagnostic efficiency of this diagnostic method. The meta-analysis results showed that c-TCD has slightly higher sensitivity and lower specificity in diagnosing PFO-RLS compared to c-TTE. The AUC values are both greater than 0.9, indicating that both c-TCD and c-TTE have high diagnostic values for PFO-RLS.

This study has the following limitations: (1) In order to determine which detection method is more accurate, we strictly followed the procedure of reviewing the article and selected case studies that use both detection techniques simultaneously, resulting in a relatively small number of included studies and patient cases; (2) Although continuous patient enrollment has been reported in most studies, selection bias cannot be completely ruled out when c-TCD is used as a trial to be evaluated, as there may be some patients with poor temporal window detection. The biggest limitation of c-TCD is poor temporal window detection, which makes it impossible for 10 to 15% of patients over 60 years old to undergo examination (36); (3) After undergoing Valsalva maneuver, the positive detection rate of PFO-RLS in patients increases. Therefore, the standardization of Valsalva maneuver has a significant impact on the detection results. The accuracy of the experiment is also indirectly affected by the standardization of the Valsalva test. Non-standardization of the Valsalva test will increase the false negative rate of the experiment results. The degree to which Valsalva maneuver increases right atrial end-diastolic pressure in various examinations may not be completely consistent, which may cause bias in the results (37). (4) The research language is limited to English and case–control studies, and there may be publication bias, selection bias, and language bias. (5) The heterogeneity of this study is high, therefore, subgroup analysis and sensitivity analysis were used. Although there is a certain relationship between c-TCD heterogeneity and age, more research is still needed, with more subgroups to confirm the reasons for heterogeneity between studies. (6) The variability of different machines and differences in contrast agents may affect the presented results. Therefore, more rigorous research is needed in the future to address the methodological limitations of these issues.

In summary, c-TCD has slightly higher sensitivity and lower specificity in diagnosing PFO-RLS compared to c-TTE. Therefore, the diagnosis of PFO requires a multidisciplinary and multi-instrument approach. The high sensitivity of c-TCD in detecting RLS should be utilized, and in positive cases, c-TTE should be used to confirm whether shunting is actually due to PFO or other anatomical conditions, such as atrial defects or pulmonary arteriovenous fistulas. Both c-TCD and c-TTE have high diagnostic value for PFO-RLS and can be used as screening methods for PFO-RLS.

## Data availability statement

The original contributions presented in the study are included in the article/Supplementary material, further inquiries can be directed to the corresponding author.

## Author contributions

DZ: Writing – original draft, Writing – review & editing. LJ: Writing – review & editing. Y-NC: Writing – review & editing. M-FP: Writing – review & editing.

## Supplementary material

SUPPLEMENTARY FIGURE S1 Percentage of included studies with the risk of bias and suitability evaluation results by QUADAS-2 tool. Green bar = “low” risk, yellow bar = “unclear” risk, and red bar = “high” risk.

SUPPLEMENTARY FIGURE S2 The assessment of risk of bias for included study. Quality is represented by colors using green (+) as yes (high quality), yellow (?) as unclear, and red (–) as no (low quality).

SUPPLEMENTARY FIGURE S3 Sensitivity analysis of studies.

SUPPLEMENTARY FIGURE S4 Funnel diagram of c-TCD and c-TTE. Panel A is the funnel diagram of c-TCD; panel B is the funnel diagram of c-TTE.

SUPPLEMENTARY FIGURE S5 Fagan diagram of c-TCD and c-TTE. Panel A is Fagan diagram of c-TCD; panel B is the Fagan diagram of c-TTE.
