# Supplementary figures and images for: The diagnostic value of contrast-enhanced transcranial Doppler and contrast-enhanced transthoracic echocardiography for right to left shunt in patent foramen ovale: a systematic review and meta-analysis
Source: Front Neurol. 2024 Aug 2;15:1447964. doi: 10.3389/fneur.2024.1447964 (PMC11327031; doi:10.3389/fneur.2024.1447964)

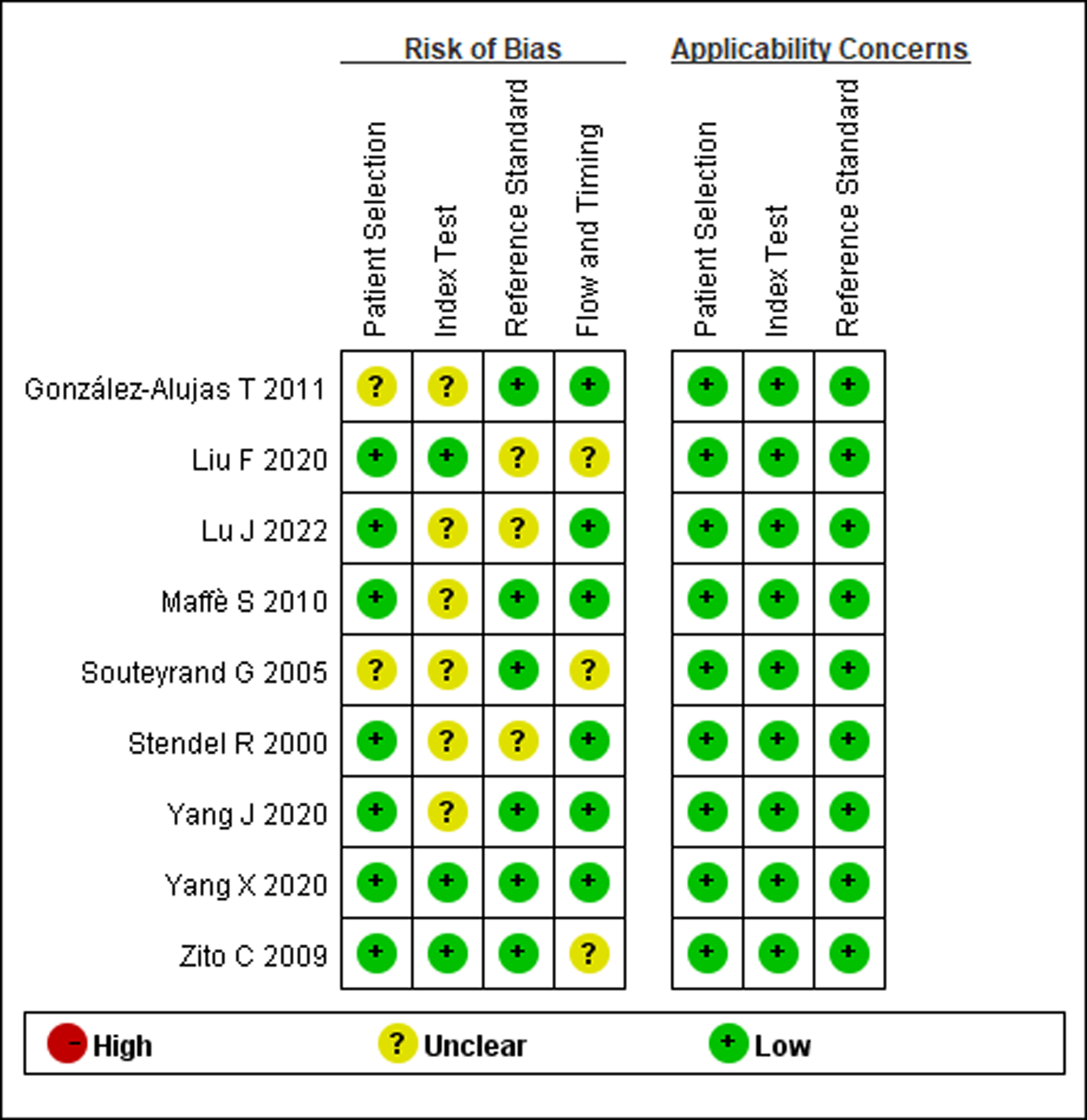

Supplement: SUPPLEMENTARY FIGURE S1 — Percentage of included studies with the risk of bias and suitability evaluation results by QUADAS-2 tool. Green bar = “low” risk, yellow bar = “unclear” risk, and red bar = “high” risk. [file Image_1.TIF]

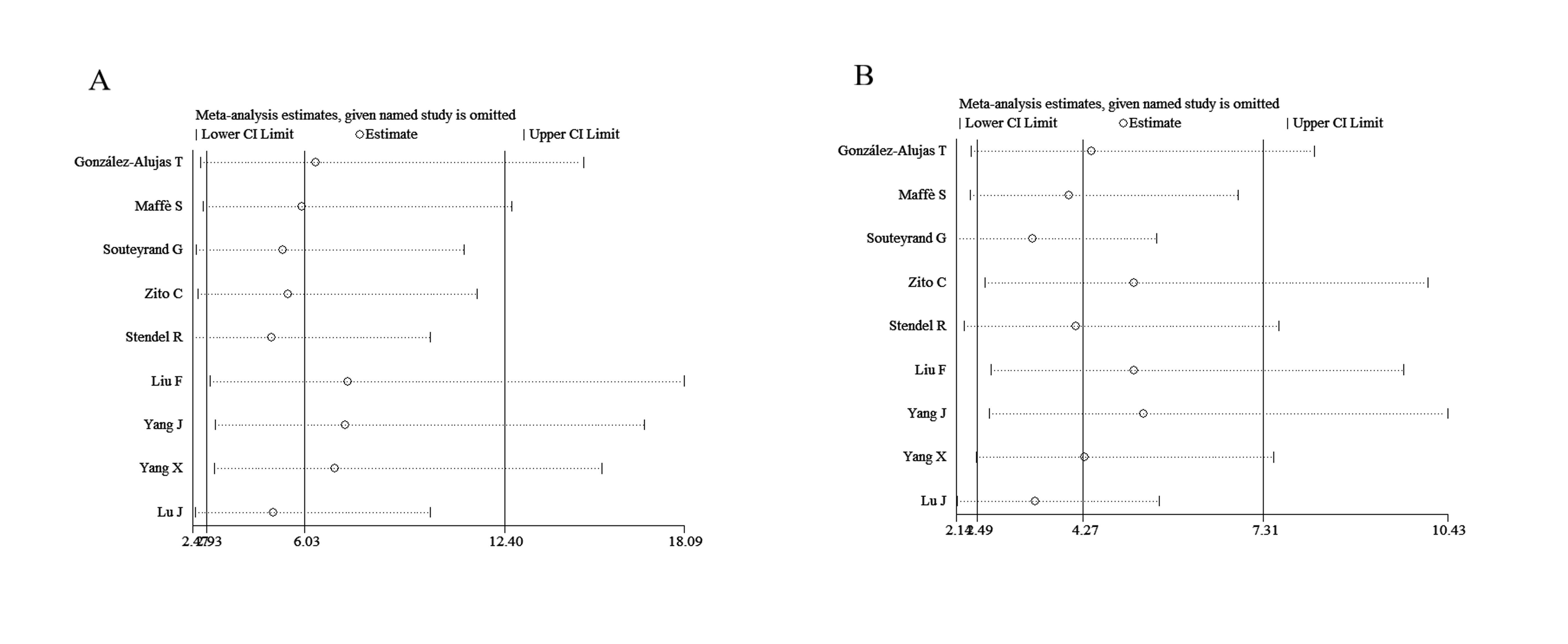

Supplement: SUPPLEMENTARY FIGURE S3 — Sensitivity analysis of studies. [file Image_3.TIF]

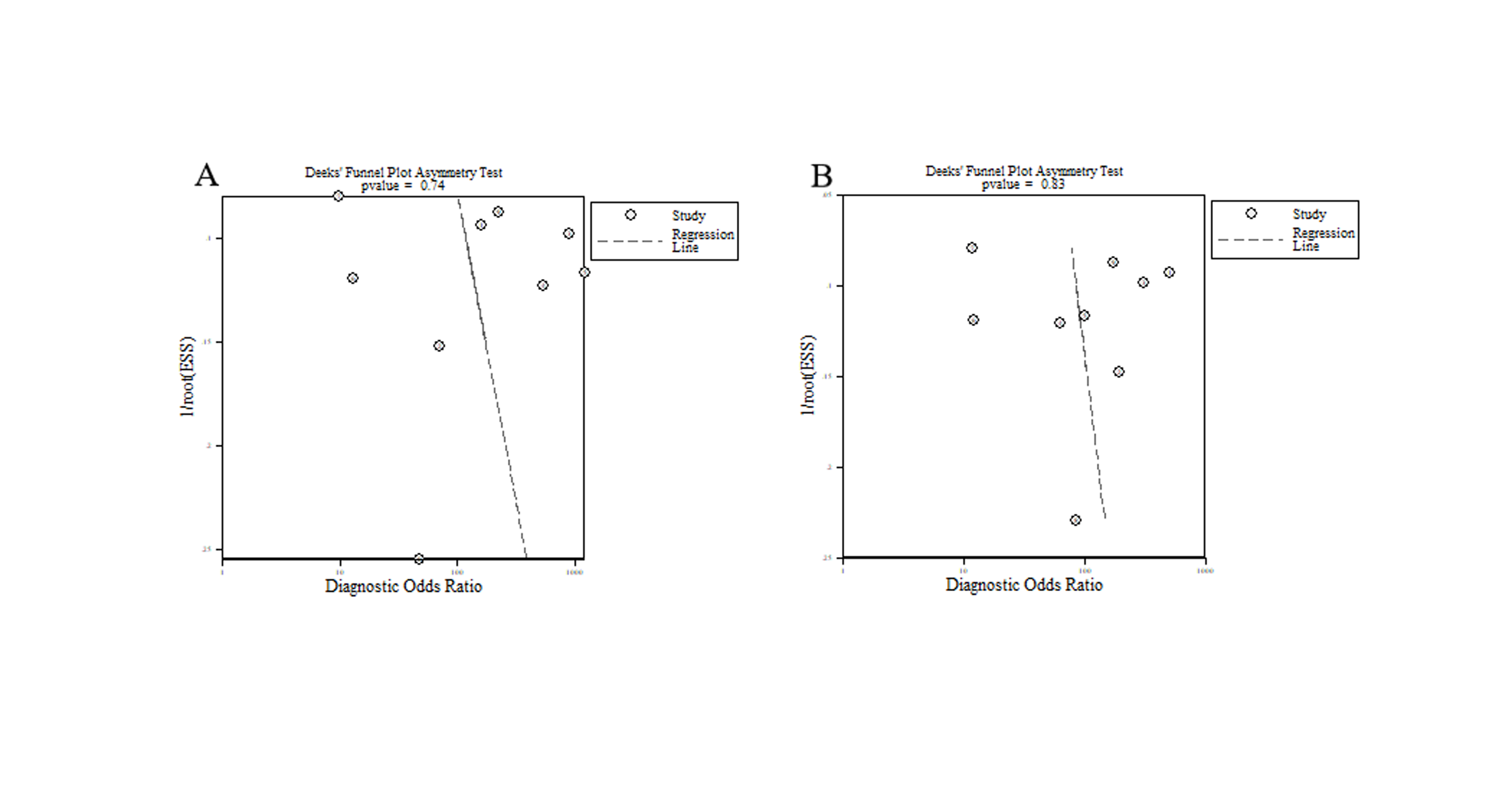

Supplement: SUPPLEMENTARY FIGURE S4 — Funnel diagram of c-TCD and c-TTE. Panel A is the funnel diagram of c-TCD; panel B is the funnel diagram of c-TTE. [file Image_4.TIF]

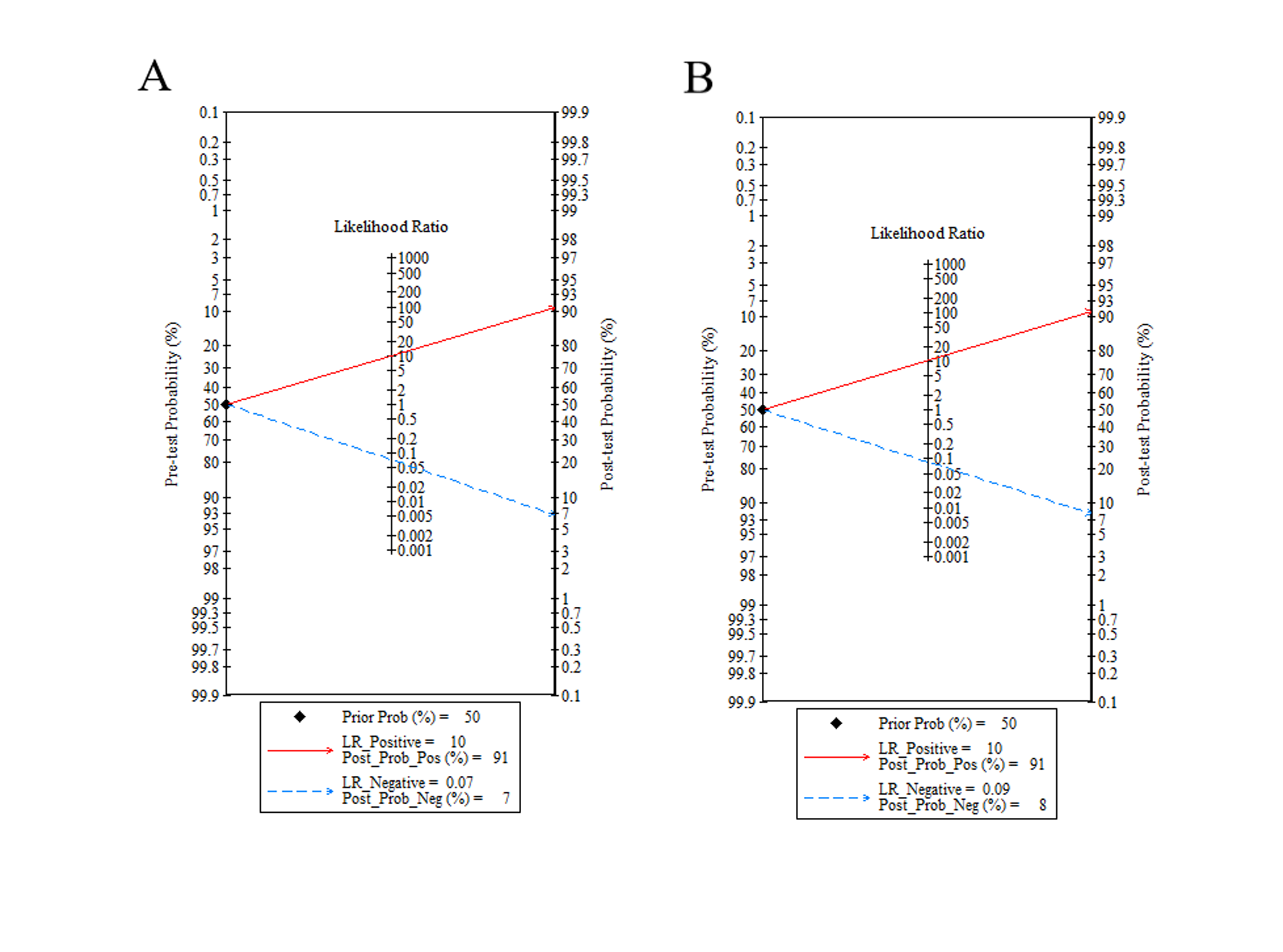

Supplement: SUPPLEMENTARY FIGURE S5 — Fagan diagram of c-TCD and c-TTE. Panel A is Fagan diagram of c-TCD; panel B is the Fagan diagram of c-TTE. [file Image_5.TIF]
